# Annotations

**Published:** 1907-06-01

**Authors:** 


### Annotations.

### The New Anti-Opium Remedy.

From Mr. L. Wray, Director of the Museums of the Federated Malay States, comes the news—welcome if the claim can be substantiated—of the discovery of an effectual cure for the opium habit. It consists of a decoction of the leaves of a climbing plant, *Combretum sundaicum,* mixed with a certain quantity of the roasted opium the smokers use. By an arrangement of doses the patient takes a daily decreasing quantity of opium and a daily increasing quantity of the *Combretum* decoction. The result, it is said, has been so successful that nearly 400 opium smokers have abandoned the habit, and the official import returns for the district where it has been used show a decrease of over 30 chests of opium per month. The discovery, we are informed, was made accidentally by some Chinese wood-cutters, who, running short of tea, made shift with the leaves of *Combretum,* and observed that it induced a distaste for the "smoke." The last word on the drug habit, whether the drug be alcohol or opium, will probably be spoken when we can substitute for these deleterious narcotics a nerve stimulant as safe as tea and coffee. And it is possible that in the *Combretum* we possess a new alkaloid having the pleasant and beneficial qualities of opium without the pains De Quincey wrote about so eloquently. As everyone knows, among the nations of North-Western Europe, although alcohol is taken with safety by a large majority of people, yet there is a certain proportion whose unstable nervous system renders them an easy prey to alcoholism. If in the new opium remedy we are fortunate to have hit upon an agent which can also satisfy or destroy the cravings of a drunkard for alcohol, then the discovery will rank as an achievement. It is to be hoped, therefore, that an immediate trial of the *Combretum sundaicum* will be made in this country, not only in the treatment of the opium habit, but also in that of chronic alcoholism.

### Professor Cushny on Drug Standardisation.

In his evidence recently given before the Royal Commission on Vivisection Professor Cushny, of University College, advances further facts in support of the contention that standardisation of certain drugs and their preparations is an absolute necessity if accurate and precise methods of treatment are to be promoted. For example, he has found that of two specimens of tincture of digitalis obtained from a firm of repute and standing the one was four times as strong as the other. Even worse is the state of matters in regard to ergot, as tests have shown that a large proportion of the drug distributed through the usual commercial channels is absolutely inert, and preparations made from such samples must, of course, be useless. Again, Dr. Cushny related from his own personal knowledge an instance in which a quantity of cannabis indica, not less indeed than 20,000 lbs., was offered to a firm of manufacturing chemists, who, before purchasing, had a specimen of the drug tested on animals, and found it entirely inactive. Such possibilities as these must force on the attention of the profession the absolute need of securing for a number of drugs in common use some official test or standard. Otherwise, it is obvious that many remedies ordered by the physician must be, without any fault or neglect on the part of the pharmacist, altogether unreliable. It is the duty of the national Pharmacopœia to secure uniformity in medicines as ordered by the physician. Pharmacologists now tell us quite definitely that in a number of drugs this can only be secured by fixing a standard based on physiological tests. Neither physical nor chemical tests are sufficient. In view of this it is manifest that the Pharmacopœia must provide for certain drugs an estimate of physiological values. Some little time ago the Therapeutical Society sent a resolution to this effect to the General Medical Council. It is essential that the dangers of the existing situation be pressed on the Council, and thus we are glad to see Professor Cushny's endorsement of the claim so presented.

### Hospital Appeals and the Central Funds.

This week we are issuing, as usual, a Special Hospital Sunday Supplement, dealing by illustrated articles with a retrospect of hospital history from the earliest times. The Hospital Sunday Fund has issued an appeal to the wealthier residents of the metropolis, in the form of an illustrated and highly-coloured card, which is forcibly written and well brings out several striking points in support of this Fund. We are convinced, however, that it is the right policy for the King's Fund and the Metropolitan Hospital Sunday Fund to abstain from issuing general appeals to the public at large, for they entail great expense, and are regarded by the hospitals as interference in the field which directly belongs to them. The general public, too, have great objections to this class of appeal by the Central Funds. The King's Fund is regarded with great confidence, and the newspapers generously afford ample opportunity to the Council to bring its claims periodically before the people who do not, it is true, at present individually subscribe annually to this Fund to the extent they undoubtedly ought to do. So far as the Sunday Fund is concerned, the issue of general appeals is apt to make people feel that Hospital Sunday as an institution is not, as it ought to be, tied up and confined to places of worship, for the Council have now gone outside the churches to approach the householders, as individuals. The effect of a general appeal in the case of the Sunday Fund may therefore prove injurious to the collections in places of worship. If so, it will be because people at large who support the hospitals through this Fund, as apart from their private gifts to individual institutions, resent a direct appeal from the central office for the reason just given. We further consider that neither fund is justified in spending money in advertising an annual subscription, except in the year in which it is given for the first time. We believe that a change of policy in this respect would tend to considerably increase the number of new annual subscribers which both funds would be glad to secure.

